# Supplementary material for: Evaluation of AlphaFold 3’s Protein–Protein Complexes for Predicting Binding Free Energy Changes upon Mutation
Source: J Chem Inf Model. 2024 Aug 8;64(16):6676–83. doi: 10.1021/acs.jcim.4c00976 (PMC11351016; doi:10.1021/acs.jcim.4c00976)
Supplement: Supplementary file 1 — ci4c00976_si_001.pdf [file ci4c00976_si_001.pdf]

Supporting Information for
Evaluation of AlphaFold 3's protein-protein complexes for predicting binding free energy changes upon mutation

JunJie Wee[1], and Guo-Wei Wei[1,2,3,*]
[1]Department of Mathematics, Michigan State University, East Lansing, MI 48824, USA
[2]Department of Biochemistry and Molecular Biology,
Michigan State University, East Lansing, MI 48824, USA
[3]Department of Electrical and Computer Engineering,
Michigan State University, East Lansing, MI 48824, USA
* Address correspondences to Guo-Wei Wei. E-mail: weig@msu.edu

Contents

S1 Validation using SKEMPI 2.0 database **2**
S1.1 Deep Learning Algorithms 2

S2 Feature descriptions **4**
S2.1 Additional details about Persistent Laplacian descriptors 4
S2.2 ESM-2 Transformer Features 6
S2.3 Harmonic Spectral Features 7
S2.4 Auxiliary descriptors 7
S2.5 Software and resources 9

S1 Validation using SKEMPI 2.0 database

The SKEMPI 2.0 database is one of the largest PPI mutation-induced binding affinity changes dataset, consisting of 7,085 entries, including both single and multi-mutations. SKEMPI 2.0 is an enhanced version of the original SKEMPI database by adding new mutations from three additional databases: AB-bind, PROXiMATE, and dbMPIKT. Existing models such as mCSM-PPI2 is validated using selected single-point mutations from SKEMPI 2.0. This validation dataset, also known as S4169, comprises of 4169 samples across 319 PPI complexes. Thereafter, a S8338 dataset was created from the S4169 set by designating the Binding Free Energy (BFE) changes of the inverse mutations as the negative equivalents of the original BFE changes induced by mutations.

A 10-fold crossvalidation is performed to validate MT-TopLap models using the S8338 dataset. Metrics such as Pearson correlation coefficient (R_p) and the root mean squared error (RMSE) are used to measure the performance of the pre-trained MT-TopLap model. For an x variable and a y variable, R_p is defined as:

$$R_p = \frac{\sum(x_i - \bar{x})(y_i - \bar{y})}{\sqrt{\sum(x_i - \bar{x})^2 \sum(y_i - \bar{y})^2}}.$$

Here, x_i is value from x variable of the ith sample and y_i is the value from y variable of the ith sample. $\bar{x}$ and $\bar{y}$ are the mean value of x and y variables respectively.

The root mean squared error (RMSE) can be calculated as follows:

$$\text{RMSE} = \sqrt{\frac{1}{n}\sum_{i=1}^{n}(y_i - \hat{y_i})^2},$$

where y_i and $\hat{y_i}$ are the predicted and truth values for the ith sample of y respectively.

S1.1 Deep Learning Algorithms

In this study, the persistent Laplacian, auxiliary, and pre-trained transformer-based features are used in the MT-TopLap model. The MT-TopLap model has 6 hidden layers and outputs the mutation-induced binding free energy changes from the 1st channel of the output layer.

MT-TopLap is a deep neural network (DNN) consists of 6 hidden layers of 15,000 neurons. The output layer's first channel is a single neuron that is fully connected with the last hidden layer and is responsible for computing predictions for S8338 dataset. It is important to note that this network is specifically designed for changes induced by mutations in the Binding Free Energy (BFE). Therefore, maintaining the consistency of all labels is crucial.

In a DNN, an optimizer is a crucial component that fine-tunes the model's parameters during the training process. Its primary role is to minimize the model's error or loss function, thereby enhancing the model's performanc A typical DNN optimizer is used to minimize the loss function

$$\underset{W,b}{\text{argmin}}\, L(W,b) = \underset{W,b}{\text{argmin}}\, \frac{1}{2}\sum_{i=1}^{N}(y_i - f(x_i;\{W,b\}))^2 + \lambda\|W\|^2,$$

where N is the number of samples, f is a function of the feature vector x_i parameterized by weight vector W and bias b. In the equation, λ refers to the penalty constant.

Standard optimization techniques for feedforward neural networks and dropout procedures are used to prevent overfitting. The structure of the network is determined through grid searches that rely on 10-fold cross-validations. The network consists of 6 layers, each containing 15,000 neurons. The hyperparameters for stochastic gradient descent (SGD) with momentum are set with momentum at 0.9, weight decay at 0, learning rate at 0.001, and batch size at 50. 200 epochs are used for the S8338 cross-validations.

S2 Feature descriptions

S2.1 Additional details about Persistent Laplacian descriptors

One of the key aspects to the success of our persistent Laplacian (PL) descriptors lies with the characterization of interactions between the mutation site and the rest of the protein. As mentioned in the Methods section, the modified distance matrix $\mathrm{D}_{\mathrm{mod}}(A_i, A_j)$ describes the distance between two atoms A_i and A_j, but excludes any interactions between A_i and A_j that are from the same subset. By constructing two types of simplicial complexes in our PL computation, such as Vietoris-Rips (VR) complex and Alpha complex, we allow our features to capture the low dimensional interactions and higher order global patterns. In addition, we incorporate element subsets $\mathcal{A}_{\mathrm{ele}}$ from elements $\{\mathrm{C}, \mathrm{N}, \mathrm{O}\}$. Here, we can denote the atoms from mutation site as $\mathcal{A}_{\mathrm{m}}(r)$ and atoms in mutation neighborhood, i.e. atoms within a distance r from mutation site, as $\mathcal{A}_{\mathrm{mn}}(r)$. The element specific subsets allow our PL descriptors to be further segmented based on different element subsets by considering one type of element in the mutation site (e.g. $\mathcal{A}_{\mathrm{C}} \cap \mathcal{A}_{\mathrm{m}}(r)$) and one other element type from mutation neighborhood (e.g. $\mathcal{A}_{\mathrm{C}} \cap \mathcal{A}_{\mathrm{mn}}(r)$).

By capturing different element/site specific combinations of interactions, the PL-based interaction features captures the similarities and differences across different PPIs. Figure S2 illustrates the C-C and O-N PL-based interaction features for AF3 predicted complex of PDB ID: 1A4Y. Rips complex representation with $\mathrm{D}_{\mathrm{mod}}$ distance function is used to generate Figure S2. In the top panel, the element/site-specific groups $\mathcal{A}_{\mathrm{C}} \cap \mathcal{A}_{\mathrm{m}}(r)$ and $\mathcal{A}_{\mathrm{C}} \cap \mathcal{A}_{\mathrm{mn}}(r)$ are used while $\mathcal{A}_{\mathrm{N}} \cap \mathcal{A}_{\mathrm{m}}(r)$ and $\mathcal{A}_{\mathrm{O}} \cap \mathcal{A}_{\mathrm{mn}}(r)$ are used for bottom panel. The parameter r in the mutation neighborhood is 4Å , i.e. 4Å from the mutation site. Essentially, Figure S2 illustrates the importance of using multiple element/site-specific groups to capture the different types of interactions based on their biophysical properties. That is the key to the success of applying topological deep learning to capture similarities and differences between wild, mutant type across different protein-protein complexes. There are 54 sets of persistent spectra in total, comprising of both harmonic and non-harmonic spectra that have the ability to understand molecular mechanism within the binding between two proteins.

In the zero-dimensional case, we consider both the harmonic and non-harmonic spectral information for each persistent Laplacian. We use a filtration process with the Rips complex and a $\mathrm{D}_{\mathrm{mod}}$ distance. The zero-dimensional PL features are created from 0 to 6 angstroms with a grid size of 0.5 angstroms.

For the non-harmonic spectral information, we count the number of non-harmonic spectra and calculate seven statistical values of non-harmonic spectra such as total, minimum, maximum, average, standard deviation, variance, and the total of squared eigenvalues. This gives us eight statistical values for each of the nine atomic pairs. As a result, the dimension of zero-dimensional PL features for a protein is 72. In total, after combining features at different dimensions for the wild type and mutant, the zero-dimensional PL-based feature size is 1872.

For one or two dimensions, we perform the filtration using the Alpha complex with the DE distance. To do this, we make use of GUDHI class “gudhi.Alphacomplex” to generate the Alpha complexes. Note that GUDHI outputs the square of the circumradius as the filtration parameter. The local protein structure’s limited number of atoms can only form a few high-dimensional

simplexes, leading to minimal shape changes. Therefore, it's sufficient to consider features from only the harmonic spectra of persistent Laplacians by encoding the topological invariants for high-dimensional interactions. Using GUDHI[1], the persistence of the harmonic spectra can be represented by persistent barcodes. The topological feature vectors are generated by calculating the statistics of bar lengths, births, and deaths. Bars shorter than 0.1 angstroms are excluded as they do not have any clear physical significance. The remaining bars are used to compute the statistics: (1) total, maximum, and average for lengths of bars; (2) minimum and maximum for the birth values of bars; (3) minimum and maximum for the death values of bars. Each set of point clouds results in a seven-dimensional vector. These features are calculated on nine single atomic pairs and one heavy atom pair. The dimension of one- and two-dimensional PL feature vectors for a protein is 140. In total, after combining features at different dimensions for the wild type, mutant, and their difference, the higher-dimensional PL-based feature size is 420.

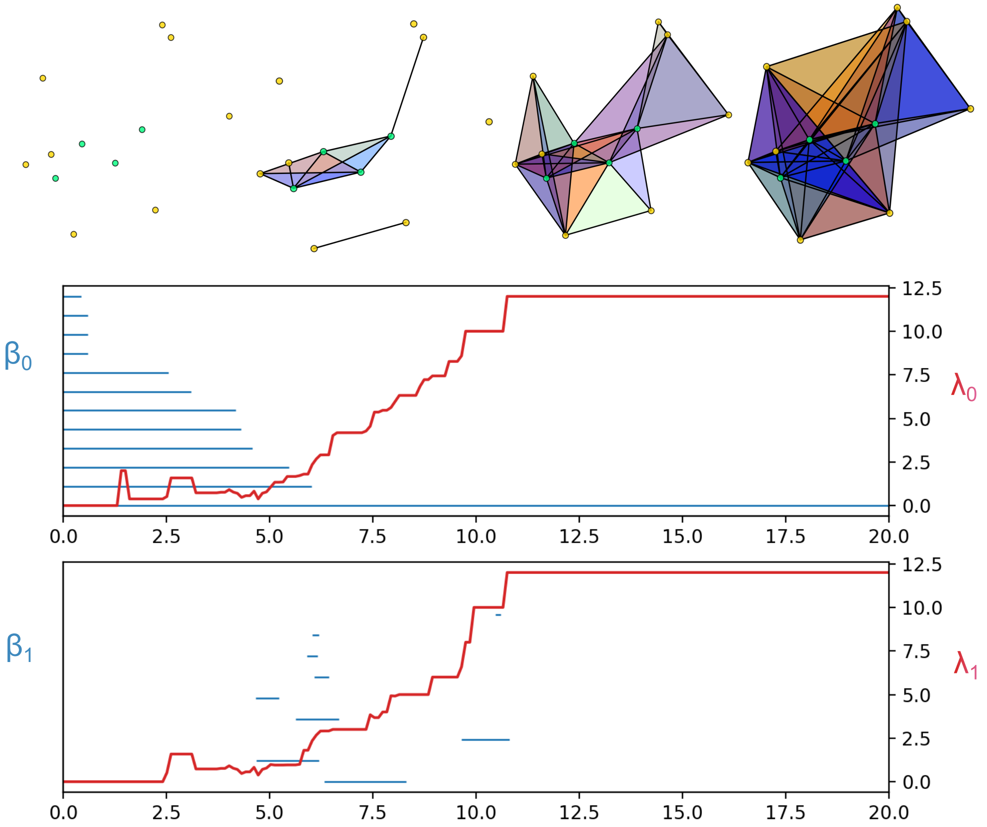

Figure S1: Comparison of persistent homology (PH) [2, 3] and persistent Laplacians (PLs) [4] for element subset C-N extracted from the mutation neighborhood of residue 435 in AF3 predicted complex of PDBID: 1A4Y [5]. The Alpha filtration characterized by the filtraton process of 12 points is shown in the top panel. The green points represent C atoms at RBD residue 435 (mutation site) while the yellow points are N atoms within 4Å from the mutation site. The corresponding topological features of dimension 0 and dimension 1 are shown the second and third panels, respectively. PH barcodes $\beta_0(r)$ and $\beta_1(r)$ are illustrated in blue. The first non-zero eigenvalues of dimension 0 ($\lambda_0(r)$) and dimension 1 ($\lambda_1(r)$) of PLs are depicted in red. The harmonic spectra of the PLs consists of all the topological invariants generated by PH, while the non-harmonic spectra of the PLs capture the additional evolution of the homotopic shape of the PLs during the filtration, which is missing from PH.

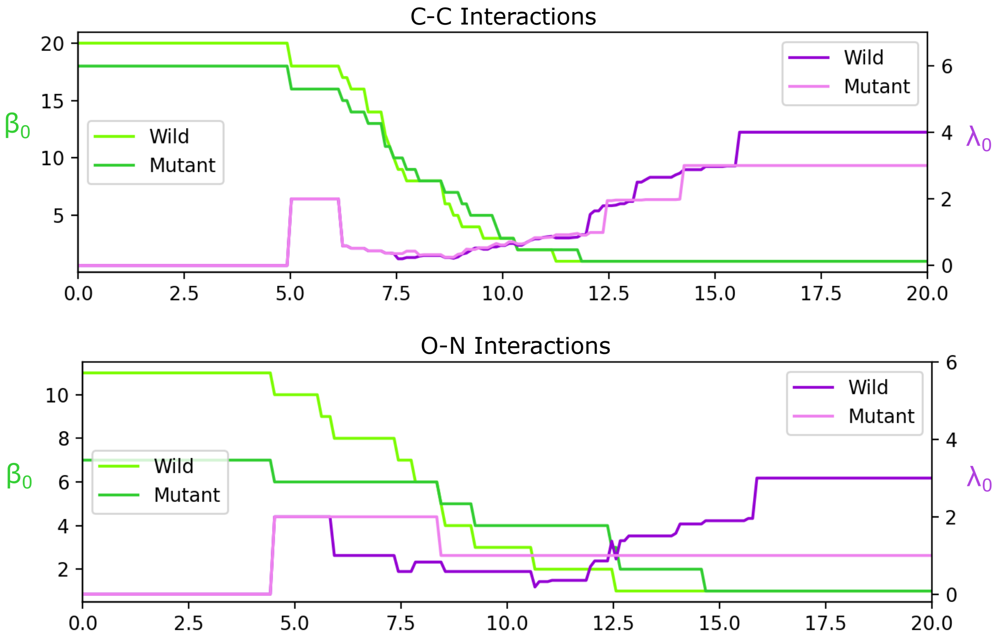

Figure S2: Top panel: An illustration of interactive PL showing C-C interactions based on D_{mod}-based filtration at residue 435 mutation site from aspartic acid to alanine in AF3 predicted complex of PDB ID: 1A4Y. Bottom panel: An illustration of interactive PL showing O-N interactions based on D_{mod}-based filtration at residue 435 mutation site from aspartic acid to alanine in AF3 predicted complex of PDB ID: 1A4Y.

S2.2 ESM-2 Transformer Features

In recent times, the field of protein property modeling has witnessed significant breakthroughs, due to rapid deployment of large language models. These models have been trained on a vast corpus of hundreds of millions of protein sequences. Notable among these early stage protein language models include ESM (Evolutionary Scale Modeling) [6] and ProtTrans[7, 8], both of which have demonstrated exceptional performance. Moreover, the effectiveness of these models has been further amplified through the use of hybrid fine-tuning methods. These methods leverage both local and global evolutionary data. The ESM model, in particular, offers the flexibility of being fine-tuned using either downstream task data or local multiple sequence alignments. In our research, we utilized the latest ESM-2 transformer[9]. This model was trained on a dataset comprising 250 million sequences. ESM-2 outperforms all tested single-sequence protein language models across a range of structure prediction tasks [9]. Inspired by AlphaFold, ESMFold which is built on ESM-2, can generate accurate structure predictions directly from the sequence of a protein [9]. In November 2022, the ESM Metagenomic Atlas was released, an open atlas of 617 million predicted metagenomic protein structures. The Atlas was updated in March 2023 in collaboration with EBI, adding another 150 million predicted structures.

The training process involved a masked filling procedure. The architecture of the ESM-2 transformer is quite impressive, consisting of 34 layers and a whopping 650 million parameters. The primary role of the ESM-2 transformer in our research is to generate sequence embeddings for both wild and mutant protein sequences. At each layer of the ESM-2, a sequence of length L was encoded into a matrix of size 1,280 x L, with the start and terminal tokens being excluded. For our

study, we used the sequence representation obtained from the final (34th) layer. We computed the average along the sequence length axis, which resulted in a vector with 1,280 components. This approach allowed us to capture the intricate details of the protein sequences effectively. This is a testament to the power and potential of large language models in advancing our understanding of complex biological systems. The ability to train these models on vast datasets and fine-tune them for specific tasks opens up major advancement for research and discovery in computational biology.

S2.3 Harmonic Spectral Features

Apart from the persistent Laplacian (PL) features, the homology groups in Persistent Homology (PH) also demonstrate the persistence of topological invariants, which also coincides with the harmonic spectra in PLs. Using site and element specific groups, we generated PH features together with PL features. The filtration process is also applied to PH in a similar way. For PH features, we generated the dimension zero features. For β_0, we performed filtration from 0 to 6Å with stepsize 0.5Å. This results in 12 intervals and 18 different barcode statistics are collected in each interval, resulting in 12×18 features. Figure S2 illustrates the difference between PH and PL features where

For each interval, the number of persistent bars are also counted, resulting in a nine-dimensional vector for each point cloud. This is performed for each of the nine element-specific groups, i.e. C-C, C-N, C-O, N-N, N-O, N-C, O-C, O-N, O-O. The dimension of PH features in total for each protein-protein complex would be 216. For dimensions one and two, similar statistics of persistent bars in PH is also calculated and used as features. All together, the PH embedding combines features at various dimensions and concatenated for the wild type, mutant PPIs, and their difference, resulting in a feature vector of length 648.

S2.4 Auxiliary descriptors

In our sophisticated MT-TopLap model, the topological descriptors elegantly weave in AF3 structural data and atom-atom interactions as topological attributes. However, it is important to note that the topological descriptors does not directly capture other crucial chemical and physical properties. To address this, we have enriched our model by incorporating a plethora of additional features. These are meticulously calculated from the chemical and physical information that we've extracted from their structural data. These additional features are primarily bifurcated into two categories: those at the atom level and those at the residue level. This comprehensive approach ensures that our MT-TopLap model is not only robust but also capable of complementing the PL descriptors from AF3 3D protein-protein complexes. This makes it a powerful tool in the analysis and understanding of complex biological systems.

S2.4.0.1 Atom-level features In auxiliary descriptors, we adopt a nuanced approach to feature generation at the atom level. Atoms are classified in various ways to ensure a comprehensive representation of features. Initially, we categorize atoms into seven distinct groups based on their types. These groups are specific to elements and include Carbon (C), Nitrogen (N), Oxygen (O), Sulfur (S), Hydrogen (H), all heavy atoms, and a group encompassing all atoms. Next, we further classify atoms based on their proximity to the mutation site. This results in three additional groups: atoms located at the mutation site, atoms near the mutation site (within a radius of 10 angstroms

from the mutation site), and a group that includes all atoms. Lastly, we take into account three different scenarios for a comprehensive analysis: the wild type, the mutant type, and the difference between them. This multi-faceted approach ensures a thorough and detailed representation of atomic-level features in our model.

For the calculation of surface areas at the atom level, we use the ESES software [10]. The areas of all atoms belonging to the same group are summed up to create a single feature, generating a total of 63 features.

When it comes to partial charges, we use the PDB2PQR software [11] in conjunction with the AMBER force field to calculate the radius and partial charge of each atom. The total of the partial charges and the total of the absolute values of the partial charges for each atomic group are considered as partial charge features, resulting in a total of 126 features.

The Coulomb energy of a single atom is calculated by adding up the pairwise Coulomb energy with all other atoms. We only consider five types of elements (Carbon, Nitrogen, Oxygen, Sulfur, and all heavy atoms) when creating Coulomb interaction features. We calculate both the Coulomb interaction energy and its absolute value, resulting in a total of 90 features.

The van der Waals energy of an atom can be calculated by adding up the pairwise Lennard-Jones potentials with all other atoms. This calculation is only considered for five types of elements: Carbon, Nitrogen, Oxygen, Sulfur, and all heavy atoms. All van der Waals interactions are computed for each atom by comparing against all heavy atoms with a cutoff of 40 angstroms, generating 45 features.

Lastly, the electrostatic solvation free energy for each atom is calculated using the Poisson-Boltzmann model with the MIBPB software [12]. The solvation free energies for all atoms within the same groups are added together, resulting in the generation of 63 features.

S2.4.0.2 Residue-type features For rest of the auxiliary descriptors, a variety of amino acids in the neighborhood of the mutation site, i.e. residues within 10 angstroms of the mutation site are used in the feature generation. Amino acids are grouped into hydrophobic, polar, positively charged, negatively charged, and special groups We calculate the count and percentage of these groups, as well as the sum, average, and variance of residue volumes, surface areas, weights, and hydropathy scores, resulting in 22 features.

Using the PROPKA software, We also calculate the pK_a shifts of seven ionizable amino acids (ASP, GLU, ARG, LYS, HIS, CYS, and TYR). The shifts are calculated as the difference of pK_a values between a wild type and its mutated type. We compute the maximum, minimum, sum, the sum of absolute values, the minimum of absolute value of total pK_a shifts, and the sum and the absolute sum of pK_a shifts based on the seven ionizable amino acid groups, resulting in 19 features.

Lastly, we use the SPIDER2 software to calculate metrics such as the probability score of mutation site residues to be coil, helix, or strand, as well as torsion angles. We also calculate these features for the wild type, the mutated type, and their difference, resulting in 12 features.

S2.5 Software and resources

- The AlphaFold Server is used to predict 317 wild-type protein-protein complexes from their protein FASTA sequences. The FASTA sequences can be obtained from https://www.rcsb.org/.
- Jackal software[13] is used to generate 8330 mutant protein-protein complexes from 317 AF3 structures. Note that we did not use AlphaFold server to predict the mutated structures. By using the Jackal software, we ensure that the 3D coordinates of the non-mutated residues remain unchanged.
- MT-TopLap model is built using PyTorch[14].
- PDB2PQR software [15] is used to calculate proteins' partial charge data. The AMBER force field is applied.
- The solvation energy and surface area information is computed using our ESES in-house online software package [16] and MIBPB [12].
- The pKa values are calculated via the PROPKA software package [17].
- The position-specific-scoring matrices (PSSM) are calculated using the ncbi-blast software [18] with an nr database. SPIDER2 software [19] calculates the secondary structure features and sequence-based torsion angle information.
- For persistent Laplacian descriptors, the GUDHI software library is used to generate both VR complexes and Alpha complexes[20] in the filtration process. The VR complexes and Alpha complexes are then used to construct the persistent Laplacian matrices. The eigenvalues are obtained by eigendecomposition.
- Structural alignment is performed by using cealign in PyMOL. Residue-based RMSD scores are also calculated after cealign is performed by taking into consideration only backbone atoms.

References

[1] T. G. Project, *GUDHI User and Reference Manual.* GUDHI Editorial Board, 2015.

[2] H. Edelsbrunner, J. Harer, *et al.*, "Persistent homology-a survey," *Contemporary mathematics*, vol. 453, no. 26, pp. 257–282, 2008.

[3] A. Zomorodian and G. Carlsson, "Computing persistent homology," in *Proceedings of the twentieth annual symposium on Computational geometry*, pp. 347–356, 2004.

[4] R. Wang, D. D. Nguyen, and G.-W. Wei, "Persistent spectral graph," *International journal for numerical methods in biomedical engineering*, vol. 36, no. 9, p. e3376, 2020.

[5] J. Lan, J. Ge, J. Yu, S. Shan, H. Zhou, S. Fan, Q. Zhang, X. Shi, Q. Wang, L. Zhang, *et al.*, "Structure of the SARS-CoV-2 spike receptor-binding domain bound to the ACE2 receptor," *nature*, vol. 581, no. 7807, pp. 215–220, 2020.

[6] A. Rives, J. Meier, T. Sercu, S. Goyal, Z. Lin, J. Liu, D. Guo, M. Ott, C. L. Zitnick, J. Ma, *et al.*, "Biological structure and function emerge from scaling unsupervised learning to 250 million protein sequences," *Proceedings of the National Academy of Sciences*, vol. 118, no. 15, p. e2016239118, 2021.

[7] A. Vaswani, N. Shazeer, N. Parmar, J. Uszkoreit, L. Jones, A. N. Gomez, Ł. Kaiser, and I. Polosukhin, "Attention is all you need," *Advances in Neural Information Processing Systems*, vol. 30, 2017.

[8] J. Devlin, M.-W. Chang, K. Lee, and K. Toutanova, "BERT: Pre-training of Deep Bidirectional Transformers for Language Understanding," in *North American Chapter of the Association for Computational Linguistics*, 2019.

[9] Z. Lin, H. Akin, R. Rao, B. Hie, Z. Zhu, W. Lu, N. Smetanin, R. Verkuil, O. Kabeli, Y. Shmueli, *et al.*, "Evolutionary-scale prediction of atomic-level protein structure with a language model," *Science*, vol. 379, no. 6637, pp. 1123–1130, 2023.

[10] B. Liu, B. Wang, R. Zhao, Y. Tong, and G. W. Wei, "ESES: software for Eulerian solvent excluded surface," *Preprint*, 2015.

[11] T. J. Dolinsky, P. Czodrowski, H. Li, J. E. Nielsen, J. H. Jensen, G. Klebe, and N. A. Baker, "PDB2PQR: Expanding and upgrading automated preparation of biomolecular structures for molecular simulations," *Nucleic Acids Res*, vol. 35, pp. W522–525, 2007.

[12] D. Chen, Z. Chen, C. Chen, W. Geng, and G.-W. Wei, "MIBPB: A software package for electrostatic analysis," *Journal of computational chemistry*, vol. 32, no. 4, pp. 756–770, 2011.

[13] J. Z. Xiang and B. Honig, "Jackal: A protein structure modeling package," *Columbia University and Howard Hughes Medical Institute, New York*, 2002.

[14] A. Paszke, S. Gross, F. Massa, A. Lerer, J. Bradbury, G. Chanan, T. Killeen, Z. Lin, N. Gimelshein, L. Antiga, *et al.*, "Pytorch: An imperative style, high-performance deep learning library," *Advances in neural information processing systems*, vol. 32, 2019.

[15] T. J. Dolinsky, J. E. Nielsen, J. A. McCammon, and N. A. Baker, "PDB2PQR: An automated pipeline for the setup of Poisson–Boltzmann electrostatics calculations," *Nucleic acids research*, vol. 32, no. suppl_2, pp. W665–W667, 2004.

[16] B. Liu, B. Wang, R. Zhao, Y. Tong, and G.-W. Wei, "ESES: Software for Eulerian solvent excluded surface," 2017.

[17] H. Li, A. D. Robertson, and J. H. Jensen, "Very fast empirical prediction and rationalization of protein pKa values," *Proteins: Structure, Function, and Bioinformatics*, vol. 61, no. 4, pp. 704–721, 2005.

[18] M. Johnson, I. Zaretskaya, Y. Raytselis, Y. Merezhuk, S. McGinnis, and T. L. Madden, "NCBI BLAST: A better web interface," *Nucleic acids research*, vol. 36, no. suppl_2, pp. W5–W9, 2008.

[19] R. Heffernan, K. Paliwal, J. Lyons, A. Dehzangi, A. Sharma, J. Wang, A. Sattar, Y. Yang,

and Y. Zhou, "Improving prediction of secondary structure, local backbone angles and solvent accessible surface area of proteins by iterative deep learning," *Scientific reports*, vol. 5, no. 1, p. 11476, 2015.

[20] C. Maria, J.-D. Boissonnat, M. Glisse, and M. Yvinec, "The GUDHI library: Simplicial complexes and persistent homology," in *Mathematical Software–ICMS 2014: 4th International Congress, Seoul, South Korea, August 5-9, 2014. Proceedings 4*, pp. 167–174, Springer, 2014.
